# Pyrophosphate of Iron

**Published:** 1862-04

**Authors:** James R. Nichols


### PYROPHOSPHATE OF IRON.

By JAMES R. NICHOLS.

Prof. Chapman's valuable paper upon the citro-ammonio-pyrophosphate of iron, published in the *Journal* a few weeks since, has awakened an unusual interest among physicians, and led to a large demand for the salt. Modern chemistry has given to the world many new compounds, some having names so truly formidable, that they are not a little puzzling to medical men, especially those whose chemistry was learned in the schools of a quarter of a century since.

It is not a matter of much surprise to find an excellent and venerable physician ordering *phyrotechnate* of iron, when we consider the unfamiliar name of the desired agent, and the haste to procure it, induced by the warm praise bestowed by Prof. C. Neither do we wonder at the inquiries of another, who wishes to know if the new sanitary pyrotechnic is not dangerous, or liable to spontaneous combustion, as *pyro* means fire, and phosphorous, a prominent constituent, can hardly be kept from setting one's saddlebags in a blaze unless protected by water.

It is important that names and therapeutic agents, should be precisely understood. Pyrophosphate of iron, correctly speaking, is the white precipitate formed when a solution of iron (2 Fe, $O_3$, $5SO_3$) is added to another of bibastic phosphate of soda ($PO_5$, 2 Na O). As a dry white powder it has been to some extent sold as pyrophosphate of iron. The salt described by Prof. Chapman, and which is inquired for as pyrophosphate of iron, bears physically no resemblance to this article, and as regards chemical constitution, varies widely from it, it being only one of its constituents. The citro-ammonio-pyrophosphate of iron, as described in the *Journal*, affords scales of beautiful light-greenish color, but if a slight amount of ammonia is added to the solution, a reddish-brown color is produced, and the dried scales are made exactly to resemble those of the citrate, or tartarate of iron. A salt of this description is the market, called pyrophosphate of iron. The medicinal effects of this preparation would in no respect differ from the other, but its physical character is so unlike, that confusion and doubt are liable to arise from similar agents existing under dissimilar forms.

It will be understood that the shops afford three distinct articles—one a dry white powder, another in brilliant green scales, and still another in red scales—all of which pass under the general designation of pyrophosphate of iron. To avoid very long names, and secure uniformity of physical aspect, I would suggest that medicinal pyrophosphate of iron be regarded as the preparation obtained by dissolving the moist white precipitate, before alluded to, in an exactly neutral solution of citrate of ammonia, or soda, and which affords scales and syrup of the elegant greenish hue. There are some apparently valid reasons why soda should be introduced into the preparation rather than ammonia.

The earthy portion of bones is essentially a tribasic phosphate of lime, and the principle of blood which affords an alkaline reaction is a tribasic phosphate of soda with two eqs. of fixed base and one eq. of basic water ($PO_5$, 2NaO HO). The chief salt in the juice of flesh and in the gastric juice, is a tribasic phosphate of potash, with one eq. of fixed base and two eqs. of basic water ($PO_5$, KO, 2HO. No one can doubt that each of these peculiar phosphates has important functions to perform in the animal economy, and their presence in abnormal quantities may be fruitful sources of disease. Soda performs a much more important part than potassa, inasmuch as it is found directly in the circulation. The principal salt in the blood to which it owes its peculiar power of absorbing and giving off carbonic acid, is a tribasic phosphate of soda, as has been stated, and therefore it may be as intimately connected with vitality and health, as iron. To what extent vital force is capable of breaking up complex salts directly administered, and forming new ones adapted to the wants of the system, is a point upon which we need information. We also need more light as regards chemical agents, or combinations best adapted to effect certain morbid conditions of the animal economy. The phosphatic salts certainly play a very important part in the chemistry of life, and that with the soda base is particularly prominent. Hence it is inferred, that when we desire to introduce a salt of phosphoric acid as a remedial agent, and it becomes necessary to associate therewith an alkali, soda or potash should be selected in preference to ammonia, as this latter is not found in the blood or tissues, and is only formed from their decomposition and decay.

In the phosphate iron salt under consideration, it would seem preferable to use soda with the citric acid in rendering the same soluable. Citrate of soda dissolves the freshly-precipitated pyrophosphate of iron as readily as the ammonia salt, and the resultant scales and syrup are equally as beautiful and tasteless.

The pyrophosphate of iron under consideration, is a sesquioxide salt, and what degree of ready assimilability it may possess, is not as yet, I presume, fully ascertained. Doubtless, it is a valuable agent, but how much more than the numerous other sesquioxide combinations of the metal, can only be learned from extensive trial. Its freedom from unpleasant taste is certainly in its favor.

When it is desirable to administer phosphorous compounds with iron, I am inclined to think a lower oxy-salt of the element, with one of the iron containing also a less amount of oxygen, is to be preferred. The hypophosphite of the protoxide of iron, in the form of syrup, is a stable compound, and possesses the least possible ferruginous taste. In the limited trials to which it has been subjected, it has proved to be remarkably prompt in its tonic and chalybeate influence, and indeed we should expect this, from what we positively know of the behavior of mineral salts under the influence of vital chemical action.

The pyrophosphate of iron, as Prof. C. suggests, is best given in the form of a thin syrup, which may be prepared from the original solution, as made ready for drying. That containing eight grains of the anhydrous, or fifteen of the combined salt, to the fluid ounce, given in teaspoonful doses, affords about the requisite amount for adults.—*Boston Med. Journal.*

